# MIR99AHG inhibits EMT in pulmonary fibrosis via the miR-136-5p/USP4/ACE2 axis

**DOI:** 10.1186/s12967-022-03633-y

**Published:** 2022-09-23

**Authors:** Jun Wang, Yuan Xiang, Sheng-Xi Yang, Hui-Min Zhang, Hui Li, Qi-Bei Zong, Le-Wei Li, Li-Li Zhao, Ruo-Han Xia, Chao Li, Le-Yuan Bao, Tong-Cun Zhang, Xing-Hua Liao

**Affiliations:** 1 Institute of Biology and Medicine, College of Life and Health Sciences, Wuhan University of Science and Technology, Hubei, 430081 People’s Republic of China; 2 Department of Medical Laboratory, Tongji Medical College, Central Hospital of Wuhan, Huazhong University of Science and Technology, Hubei, 430014 People’s Republic of China; 3 Yangtze University Health Science Center, Hubei, 430014 People’s Republic of China; 4 Department of Clinical Neurosciences, University of Cambridge, Cambridge, UK; 5 Department of Applied Mathematics and Theoretical Physics, University of Cambridge, Cambridge, UK

**Keywords:** MIR99AHG, Lung adenocarcinoma, miR-136-5p, USP4, ACE2, Fibrosis

## Abstract

**Background:**

Long non-coding RNAs (lncRNAs) are closely related to the occurrence and development of cancer. Abnormally expressed lncRNA can be used as a diagnostic marker for cancer. In this study, we aim to investigate the clinical significance of MIR99AHG expression in lung adenocarcinoma (LUAD), and its biological roles in LUAD progression.

**Methods:**

The relative expression of MIR99AHG in LUAD tissues and cell lines was analyzed using public databases and RT-qPCR. The biological functions of MIR99AHG were investigated using a loss-of-function approach. The effect of MIR99AHG on lung fibrosis was assessed by scratch assay, invasion assay and lung fibrosis rat model. FISH, luciferase reporter assay and immunofluorescence were performed to elucidate the underlying molecular mechanisms.

**Results:**

LncRNA MIR99AHG expression level was downregulated in LUAD tissues and cell lines. Low MIR99AHG levels were associated with poorer patient overall survival. Functional analysis showed that MIR99AHG is associated with the LUAD malignant phenotype in vitro and in vivo. Further mechanistic studies showed that, MIR99AHG functions as a competitive endogenous RNA (ceRNA) to antagonize miR-136-5p-mediated ubiquitin specific protease 4 (USP4) degradation, thereby unregulated the expression of angiotensin-converting enzyme 2 (ACE2), a downstream target gene of USP4, which in turn affected alveolar type II epithelial cell fibrosis and epithelial–mesenchymal transition (EMT). In summary, the MIR99AHG/miR-136-5p/USP4/ACE2 signalling axis regulates lung fibrosis and EMT, thus inhibiting LUAD progression.

**Conclusion:**

This study showed that downregulated MIR99AHG leads to the development of pulmonary fibrosis. Therefore, overexpression of MIR99AHG may provide a new approach to preventing LUAD progression.

**Supplementary Information:**

The online version contains supplementary material available at 10.1186/s12967-022-03633-y.

## Background

Pulmonary fibrosis is a chronic progressive disease usually leading to respiratory failure of the lung and patient’s death within 3–5 years after diagnosis [1]. The pathogenesis of pulmonary fibrosis involves the secretion of multiple fibrogenic growth factors and cytokines by the damaged type II alveolar epithelial cells, which may cause subsequent downstream pathophysiological processes, including epithelial–mesenchymal transition (EMT), recruitment and activation of fibroblasts, formation of fibroblast foci, and production of large amounts of extracellular matrix, whose excessive deposition eventually causes pulmonary fibrosis [2]. In particular, EMT plays a central role in developing fibrosis in many organs, including the lung [3–5]. Lung cancer frequently occurs in patients with long-term chronic pulmonary fibrosis. Although surgery plays an important role in the management of non-small cell lung cancer, this procedure cannot alleviate the impaired lung function and pulmonary fibrosis [6, 7].

Long non-coding RNAs (lncRNAs) are non-coding RNAs greater than 200 nucleotides in length [8]. A growing body of evidence suggests that lncRNAs play an important role in cell differentiation and development by controlling gene expression at the transcriptional, post-transcriptional and epigenetic levels [9]. Recent studies have revealed that the expression of lncRNAs is dysregulated in various cancers, such as breast cancer, liver cancer, gastric cancer [10–12]. This finding highlights the potential of lncRNAs as diagnostic and prognostic biomarkers for cancer.

LncRNA MIR99AHG is a long non-coding RNA located at 21q21.1 with 16 transcripts, transcribed as a polycistronic miRNA host gene that produces spliced lncRNAs and three intron microRNAs (miRNAs): hsa-miR-99A, hsa-miR-125B2, and hsa-let-7C [13]. LncRNA MIR99AHG was firstly identified in acute megakaryocytic leukemia in 2014 and was reported upregulated in acute myeloid leukemia [14]. Additionally, LncRNA MIR99AHG was found to promote gastric cancer progression by inducing EMT and inhibiting apoptosis through the hsa-miR-577/FOXP1 axis [15]. Therefore, LncRNA MIR99AHG could serve as an effective prognostic marker. However, no previous study has addressed the role of LncRNA MIR99AHG in pulmonary fibrosis.

Ubiquitin specific proteases (USPs) exert various functions in tumorigenesis, including enhancing EMT, maintaining stemness, promoting metastasis and proliferation, and regulating tumor microenvironment and DNA damage repair [16]. Ubiquitin-specific protease 4 (USP4) is one of the best-studied USP family, regulating various signalling pathways by deubiquitinating key proteins. Previous studies have found that USP4 can inhibit p53 transcription and pro-apoptotic functions by stabilizing HDAC2 [17], whereas the deubiquitinase USP4 stabilizes Twist1 protein to promote lung cancer stemness [18]. Further, USP4 expression may independently predict favorable survival in LUAD [19]. Therefore, USP4 may contribute to the progression of pulmonary fibrosis, which, however, has not yet been studied.

Angiotensin-converting enzyme 2 (ACE2) is a type I membrane protein widely expressed in lungs, heart, kidney and intestine [20, 21]. Positive factors dominated by the angiotensin-converting enzyme (ACE)/angiotensin II (AngII)/AngII type 1 receptor (AT1R) axis have been reported to be activated during pulmonary fibrosis and EMT, while negative regulators dominated by the ACE2/angiotensin 1–7 (Ang1–7)/Mas axis are reduced during fibrosis and EMT [22]. AngII has been reported to significantly promote pulmonary fibrosis progression, and ACE2 obtains large amounts of AngII by hydrolyzing Ang-(1–7) to reduce local aggregation of pro-fibrotic factors in fibrotic lesions and thus inhibit the progression of pulmonary fibrosis [23].

In this study, we hypothesized that MIR99AHG plays an important role in pulmonary fibrosis based on the previous findings. Specifically, we investigated the effects and mechanisms of MIR99AHG on EMT involved in fibrosis in LUAD. Our results show that MIR99AHG is significantly downregulated in LUAD and acts as an independent predictor of patient survival. Furthermore, our mechanistic studies suggest that MIR99AHG may regulate ACE2 expression by acting as a competitive endogenous RNA (ceRNA), which inhibits the degradation of USP4 mRNA, and thus ACE2 mRNA, by competing with miR-136-5p. Our findings suggest that MIR99AHG is a potential EMT inducer and could serve as a therapeutic target for pulmonary fibrosis.

## Materials and methods

### Cell lines

Human renal epithelial cell line HEK293T, human bronchial epithelial cell line BEAS-2B and human adenocarcinoma cell line A549 were purchased from Beijing Beina Chuanglian Institute of Biotechnology (Beijing, China). They were cultured in DMEM (PWL003-DZ, meilunbio, Wuhan, China) or 1640 medium (PWL047, meilunbio, Wuhan, China) supplemented with 10% fetal bovine serum (FBS) in a 5% CO_2_ incubator.

### Patients and tissue samples

Fresh tumor tissues and paired adjacent non-tumor samples were collected from 20 LUAD patients (8 females, aged 37‒69 years with a mean age of 52.4 years) who underwent surgical resection at the Hubei Cancer Hospital from October 2018 to December 2020. Twenty patients had a history of pulmonary fibrosis. All patients were diagnosed by two independent pathologists. The histopathological characteristics of LUAD tumors were assessed according to the 8th edition of the American Joint Committee on Cancer (AJCC) staging system. Tissue samples were preserved in liquid nitrogen. All patients did not receive chemotherapy or radiotherapy before surgery and signed a written informed consent. The study was approved by the ethical review committee of Hubei Cancer Hospital.

### Plasmid construction and cell transfection

Find the target gene sequence according to the UCSC website (https://genome.ucsc.edu/); primers were designed, and the sequences were synthesized by Beijing Tsingke Biotechnology Co., Ltd. The total RNA of 293 cells was extracted and reverse transcribed to generate cDNA. Using the cDNA as a template, the optimal PCR reaction conditions were used to amplify the target gene sequence. The amplified product was subjected to 1% agarose gel electrophoresis, gel-tipped, purified and recovered. Dicer digests the plasmid vector, mixes the target fragment with the plasmid vector in an appropriate ratio, and reacts with homologous recombinase overnight. The ligation product was transformed into DH5α competent cells, and the transformed product was inoculated on an agar plate screened by ampicillin, 37 ℃ 5% CO_2_, incubated in an incubator for 12 h, and a number of colonies grew on the agar plate, and 10 positive clones were picked to prepare bacterial fluid, PCR was performed to identify gene expression to determine whether the transfection was successful. For sequencing analysis, five bacterial solutions identified by PCR and successfully transfected were selected and synthesized by Beijing Tsingke Biotechnology Co., Ltd. Cells were cultured to 50–60% density before being transfected. All plasmids, mimic, and inhibitors were transfected with lipofectamine 3000 (L3000008, Thermo Fisher Scientific, Massachusetts, USA) according to the manufacturer’s instructions. All plasmid construction primer sequences are listed in Additional file 1: Table S1.

### Fluorescence in situ hybridization (FISH)

Specific FISH probes targeting MIR99AHG and miR-136-5p were designed and synthesized by Servicebio (Wuhan, China). Histological sections were baked at 65 ℃ overnight, dewaxed in xylene at room temperature twice, 10 min each, then immersed in 100% ethanol for 5 min, rehydrated and treated with 30% acidic sodium sulfite at 50 ℃ for 20–30 min, 2×SSC (saline sodium citrate) was rinsed twice, 5 min each, soaked in proteinase K working solution, incubated at 37 ℃ for 7–8 min. After rinsing with 2×SSC solution, dehydrated in 70%, 85%, and 100% ethanol pre-cooled at minus 20 ℃ for 2 min each, soak in acetone for 2 min, dry, add coverslip to 56 ℃ for 3 min. Soak the slide in denaturing solution for 5 min, gradient dehydration in pre-cooled 70%, 85%, and 100% ethanol for 3 min each, preheated at 45 ℃ to 50 ℃ for 2–5 min, and hybridized with the probe overnight in a 42 ℃ incubator. The slides were washed with formamide, dried naturally in the dark and counterstained with diamidinophenyl indole fluorescent dye (DAPI), and placed in the dark. All images were analyzed on a confocal laser scanning microscope (Olympus, Tokyo, Japan). The FISH probe sequences are shown as follows: MIR99AHG: 5′-CTCTTCTTTGTGTAGCGGAGACGACTGTGGTTGAT-3′; miR-136-5p: 5′-CCATCATCAAAACAAATGGAGT-3′.

### Western blotting (WB)

Cells were lysed with RIPA lysis buffer (MA0151, meilunbio, Wuhan, China) containing protease inhibitor on ice. Protein extracts (30 µg) were separated by electrophoresis on SDS-polyacrylamide gels, then transferred to PVDF membrane, blocked with milk for 1 h at room temperature, and then incubated with antibodies overnight. Wash three times using TBST. Add secondary antibody (1:5000, AS014, ABclonal, Wuhan, China) and incubate at room temperature for 1 h. Use TBST to wash after imaging on a chemiluminescence imaging system (Thermo Fisher Scientific, Massachusetts, USA). The following antibodies were used: Anti-GAPDH (1:1000, AC002, ABclonal, Wuhan, China), Anti-TGF-β1 (1:1000, A21244, ABclonal, Wuhan, China), Anti-ACE2 (1:1000, AC002, ABclonal, Wuhan, China), Anti-USP4 (1:1000, A20005, ABclonal, Wuhan, China), Anti-COL1A1 (1:1000, A16891, ABclonal, Wuhan, China), Anti-ACTA2 (1:1000, A1011, ABclonal, Wuhan, China), Anti-Vimentin (1:1000, A19607, ABclonal, Wuhan, China), Anti-E-Cadherin (1:1000, A20798, ABclonal, Wuhan, China).

### RT-qPCR analysis

Expression levels of MIR99AHG and other genes in LUAD tissues and cells were measured using qRT-PCR according to the manufacturer’s instructions (11123ES10, Yeasen, Shanghai, China). GAPDH was used as a control. Primers are listed in Additional file 1: Table S2. Total RNA was extracted using an ultra-pure RNA kit (CW0581, CWBIO, Beijing, China) and cDNA was synthesized using a HiScript II Q RT SuperMix for qPCR kit (R222-01, Vazyme, Nanjing, China).

For miRNA quantification, Bulge-loop™ miRNA qRT-PCR primer sets were designed by Beijing Tsingke Biotechnology Co., Ltd. The cDNA was synthesized using the miRNA 1st Strand cDNA Synthesis Kit (MR101-01, Vazyme, Nanjing, China ).

RT-qPCR was performed with the cDNA obtained in the above steps as a template, and three replicate wells were set in each group. PCR was performed for 40 cycles of 5 min at 95 ℃, 12 s at 95 ℃, 20 s at 58 ℃ and 20 s at 72 ℃.

### Lentiviral transfection and stable cell line construction

The lentiviral packaging of small hairpin RNA (shRNA) overexpressing MIR99AHG and targeting miR-136-5p was performed in HEK293T cells. After 72 h, the collected lentivirus was transfected into A549 cells with 6 µg/mL polybrene for 48 h. Stable cell clones were selected using puromycin (1.5 µg/mL) for 1 week. Overexpression or knockdown efficiency was confirmed by RT-qPCR.

### Luciferase reporter experiment

The transfected cells were lysed with 1× passive lysis buffer on ice for 20 min. The supernatant was added to luciferase assay buffer after centrifugation at 4 °C to determine the fluorescence values. The same volume of supernatant was taken for protein quantification, and finally, the relative luminescence values were obtained by normalizing the measured luminescence values for each group and the corresponding total protein content.

### Cell migration and invasion

Scratch assay was used to test the cell migration ability. A total of 2 × 10^5^ cells were inoculated into 6-well plates. Cells were transfected according to the cell transfection protocol. When the cell density reached 90%, the cell layer was scratched with a 200 µL pipette tip, the cells were washed three times with 1× PBS, and the culture was continued with a medium containing 20% FBS. At different time points, images of the plates were acquired using a microscope (Sanshen, Shanghai, China) and the relevant areas of the wounds were acquired using Image J software to quantify and calculate the cell scratch area.

For the invasion assay, 1 × 10^5^ transfected cells were placed in the invasion assay upper chamber lined with matrigel and a medium containing 20% serum was added to the lower chamber. The cells were fixed with 4% paraformaldehyde after 24 h incubation, and medical swabs were used to wipe off the cells in the upper chamber. Cells that had migrated or invaded the membrane were fixed with methanol, stained with 0.1% crystal violet for 25 min, and photographed under an inverted microscope.

### Hematoxylin–eosin staining and immunohistochemistry experiment

The tumor tissues of the nude mouse were sent to Servicebio Biotech for paraffin-embedded sectioning. Hematoxylin–eosin staining kit (G1120, Solarbio, Beijing, China) is used for hematoxylin–eosin staining (H&E). Use streptavidin alkaline phosphatase (SABC-AP) immunohistochemical staining kit (ab64261, Abcam, Cambridge, UK) for immunohistochemistry (IHC) experiments. Sections were de-paraffinized, rehydrated, antigens were retrieved with sodium citrate antigen retrieval buffer (pH 6.0), and then endogenous peroxidase was inactivated with 3% hydrogen peroxide for 20 min at room temperature. After washing, nonspecific antigen binding was blocked with 10% normal goat serum for 30 min at 37 °C, incubated with primary antibody overnight at 4 °C, and then incubated with secondary antibody for 45 min at room temperature. Chromogenic detection was performed using 3,3-diaminobenzidine (DAB) chromogenic kit (G1212-200T, Servicebio, Wuhan, China). Nuclei were counterstained with hematoxylin. All the above operations are carried out according to the manufacturer’s instructions.

### Co-immunoprecipitation (Co-IP)

A549 cells were lysed with 1 mL IP lysis buffer for 30 min on ice, then centrifuged at 13,000×*g* for 10 min at 4 ℃. Add 10 µL of supernatant to 2×SDS loading buffer and denature at 95 ℃ for 10 min before use. Pretreated protein A/G magnetic beads (Bimake, Shanghai, China) containing 500 µL of RIPA were added to 5 µg of antibody, followed by incubation at 4 ℃ for 30 min. The mixture was placed on a magnet for 1 min, the supernatant was removed, and washed three times. Beads were resuspended with sample (500 µL) and incubated overnight at 4 ℃ with rotation. The mixture was placed on a magnet for 1 min, the supernatant was removed, and washed three times. Magnetic beads were added to 1× SDS loading buffer, denatured at 95 ℃ for 10 min, centrifuged at 12,000×*g* for 10 min, and the supernatant was saved and assessed by WB.

### Immunofluorescence

Treated A549 cells were fixed with 4% paraformaldehyde for 30 min and permeabilized by 0.5% Triton X-100 for 30 min, followed by blocking in 3% BSA for 30 min. Cells were then incubated overnight at four degrees with primary antibodies (1:100, A1011, ABclonal, Wuhan, China), anti-COL1A1 (1:100, A16891, ABclonal, Wuhan, China). Fluor^®^ 488 (1:100, ab150077, Abcam, Cambridge, UK) was then incubated with Alexa-conjugated IgG for 1 h. Nuclei were stained with DAPI. Cells were observed by laser confocal microscopy (Olympus, Tokyo, Japan).

### Biotin-labeled miRNA pull-down

Cell lysates were harvested 48 h after transfection with 50 nM biotin-labeled miRNA (miR10000448, RiboBio, Guangzhou, China). Streptavidin-conjugated Dynabeads (S11223, Thermo Fisher Scientific, Massachusetts, USA) were washed and resuspended in buffer. An equal volume of biotin-labelled miRNA was then added to the buffer. After 10 min incubation at room temperature, the coated beads were detached with a magnet for 2 min and washed 3 times. The isolated RNA was then subjected to RT-qPCR analysis.

### Bleomycin-induced murine lung fibrosis model

The protocol was approved by the Institutional Committee for Animal Care and Use of Wuhan University of Science and Technology. Pulmonary fibrosis rats model were subjected to a standard protocol as previously described. SD (Sprague Dawley) rats were anesthetized by intraperitoneal injection of 1% sodium pentobarbital (40 mg/kg), then fixed on the rat board in a supine position, the neck hair was cut off, the skin was sterilized with iodophor + 75% ethanol, and a 1 cm-long mid-neck incision was made under aseptic operation. Separate the exposed trachea layer by layer, and use curved ophthalmic forceps to pass under the trachea, slightly lift the trachea, but do not affect airway ventilation. A 1 mL syringe needle was used to puncture the two cartilage rings under direct vision, keeping the needle in the same direction as the airway. The bleomycin group was given a one-time rapid bolus injection of bleomycin 0.2 mL (5 mg/kg) into the trachea. Immediately pull out the needle, erect the rat plate, keep the rat in an upright position, and rotate it back and forth for 1–2 min, so that the liquid reaches the lungs on both sides as much as possible and distributes it evenly. SD rats were housed under standard conditions with free access to water and rodent laboratory food. Lentivirus injection in SD rats 28 days after induction with bleomycin. SD rats were divided into two groups and injected intraperitoneally with control lentivirus and lentivirus encoding MIR99AHG, respectively. All SD rats were euthanized 30 days after lentivirus injection, and then lung tissues were taken for Masson staining and H&E staining.

### Statistical analysis

All statistical analyses were performed using GraphPad Prism. All experiments were repeated 3 times, and the data were expressed as mean ± SD. Differences between various groups were evaluated using a two-tailed Student’s t-test. The survival difference between patient groups was tested using the log-rank test and plotted in Kaplan–Meier survival curves. Pearson correlation test was used to analyze the correlations between the levels of MIR99AHG, miR-136-5p, USP4 and ACE2. A bilateral *P* < 0.05 was considered statistically significant.

## Results

### MIR99AHG inhibit LUAD progression

**Fig. 1:**
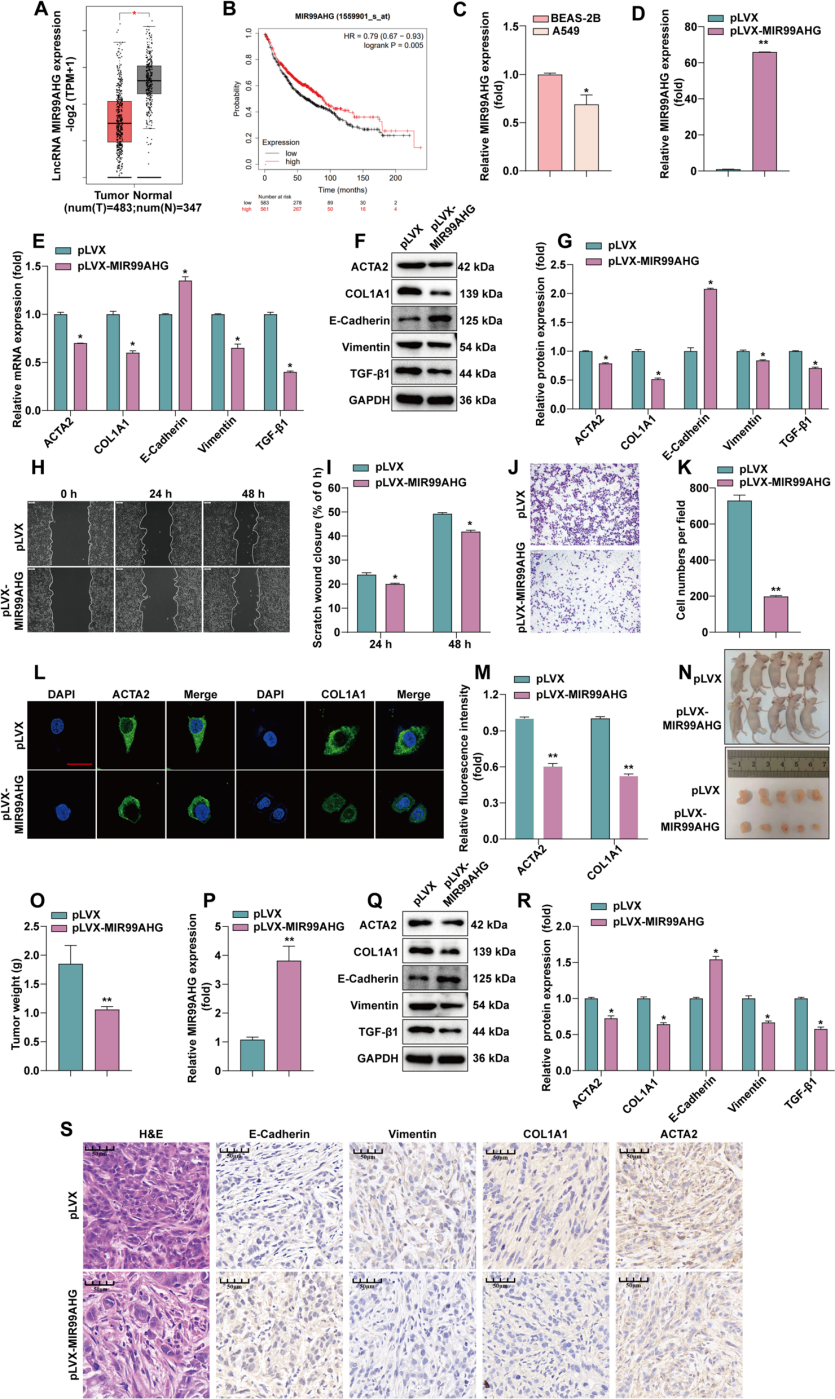
Overexpression of MIR99AHG suppresses EMT and fibrosis.** A** Analysis of LncRNA MIR99AHG expression in LUAD and normal lung tissues using the GEPIA database. **B** Analysis of the relationship between LncRNA MIR99AHG expression and survival of LUAD patients using the Kaplan–Meier plotter. **C** RT-qPCR detection of LncRNA MIR99AHG expression. **D** RT-qPCR to detect the stable overexpression efficiency of LncRNA MIR99AHG. **E** RT-qPCR to detect the expression of E-Cadherin, Vimentin, ACTA2 and COL1A1 after overexpression of LncRNA MIR99AHG. **F**, **G** The protein expression of TGF-β1, E-Cadherin, Vimentin, ACTA2 and COL1A1 was detected by WB and statistically quantified by ImageJ software. **H**, **I** After overexpression of LncRNA MIR99AHG, cell migration was monitored by wound scratch assay. Statistical data are shown. **J**, **K** After overexpression of LncRNA MIR99AHG, cell invasion was measured by invasion assay. Cell invasion was determined by invasion assay and counted by ImageJ counting. **L**, **M** Immunofluorescence assay was performed to detect the expression of fibrosis-related proteins after overexpression of LncRNA MIR99AHG. Statistical data are shown. **N** Subcutaneous tumorigenesis demonstration in nude mouse. **O** Tumor weights were represented as the means of tumor weights ± SD. **P** RT-qPCR to confirm the overexpression of LncRNA MIR99AHG in tumor tissues. **Q**, **R** WB to detect the expression of TGF-β1, E-Cadherin, Vimentin, ACTA2 and COL1A1 in MIR99AHG stabled overexpression tumor tissues. Statistical data are shown. **S** The tumor sections underwent IHC staining and H&E staining. *P < 0.05, **P < 0.01

Firstly, to compare the expression of LncRNA MIR99AHG in LUAD and normal lung tissues, we used the GEPIA database (http://gepia.cancer-pku.cn), which has profiled 483 cases of LUAD patients and 347 control specimens. Figure 1A shows that MIR99AHG expression was low in LUAD compared to normal lung tissue. The Kaplan–Meier plot (*P* = 0.035, Fig. 1B) shows that the lower level of MIR99AHG expression, dichotomized using median expression, was significantly associated with worse overall survival (OS) of LUAD patients.

We performed H&E and Masson staining on the collected 20 pairs of tumor-normal tissue to determine the feature of pulmonary fibrosis combined with LUAD (Additional file 2: Figure S1A and B). To validate the results derived from the GEPIA database, we examined the expression of MIR99AHG in the 20 pairs of specimens, which was significantly reduced in LUAD tissues (Additional file 2: Figure S1C). The FISH assay confirmed the downregulation of MIR99AHG in LUAD tissues compared to the paired non-tumor samples (Additional file 2: Figure S1D), mainly located in the cytoplasm (Additional file 2: Figure S1E). In addition, we performed RT-qPCR to quantify the expression levels of MIR99AHG in the normal human lung cell line BEAS-2B and the LUAD cell line A549. As shown in Fig. 1C, MIR99AHG has lower expression in the LUAD cell line compared to the normal cell line.

We further assessed the function of MIR99AHG in LUAD in the A549 cells expression with MIR99AHG stably overexpressed. The mRNA and protein of overexpressing MIR99AHG cells were extracted, and the expression of TGF-β1 protein, EMT-related proteins E-Cadherin, Vimentin and fibrosis markers α-smooth muscle actin (ACTA2) and collagen type I α1 chain (COL1A1) were detected. Compared with the control group, the RNA and protein expression of E-Cadherin was elevated after overexpression of MIR99AHG. In comparison, the expression of TGF-β1, Vimentin, and ACTA2 and COL1A1 were decreased at both RNA and protein levels (Fig. 1E and G). Further, the inhibitory effect of MIR99AHG overexpression on A549 cell invasion was confirmed by wound scratch and invasion assays (Fig. 1H–K). Immunofluorescence results showed that overexpressing MIR99AHG was associated with decreased expression levels of ACTA2 and COL1A1, and reduced ACTA2 fibrotic filaments with shortened length. Similarly, COL1A1 deposition was also reduced (Fig. 1L). We selected forty fields of view for statistical quantification (Fig. 1M).

To determine the in vivo effect of MIR99AHG on LUAD growth, A549 cells with a lentiviral vector producing overexpression of MIR99AHG were inoculated into the dorsal region of the mouse. The lentivector-infected A549 cells carrying control pLVX were inoculated into the dorsal region of the same mouse as control (Fig. 1N). When the tumors grew to the appropriate size, the intact tumor blocks were removed and weighed. The results (Fig. 1O) show that the tumors from the cell group with stable overexpression of MIR99AHG were less in size and weight than the control group.

The expression of MIR99AHG in tumor tissues was examined by RT-qPCR, which showed that MIR99AHG remained stably overexpressed in nude mice (Fig. 1P). The WB showed that, compared to the control group, tumor tissues with stable overexpression of MIR99AHG had decreased expression of the TGF-β1, Vimentin, and ACTA2 and COL1A1. In comparison, the expression of E-Cadherin were elevated (Fig. 1Q and R).

We performed a histopathological analysis of nude mouse tumors using H&E and IHC staining of E-Cadherin, Vimentin, ACTA2 and COL1A1. The nuclei of the MIR99AHG overexpression group showed the significant feature of crinkling and unclear cell contours. We found that increased expression of E-Cadherin, while decreased expression of Vimentin, ACTA2 and COL1A1, and fibrosis markers (Fig. 1S), suggesting that overexpression of MIR99AHG may inhibit the progression of EMT and fibrosis.

### MiR-136-5p bind to MIR99AHG and competes with its expression

**Fig. 2:**
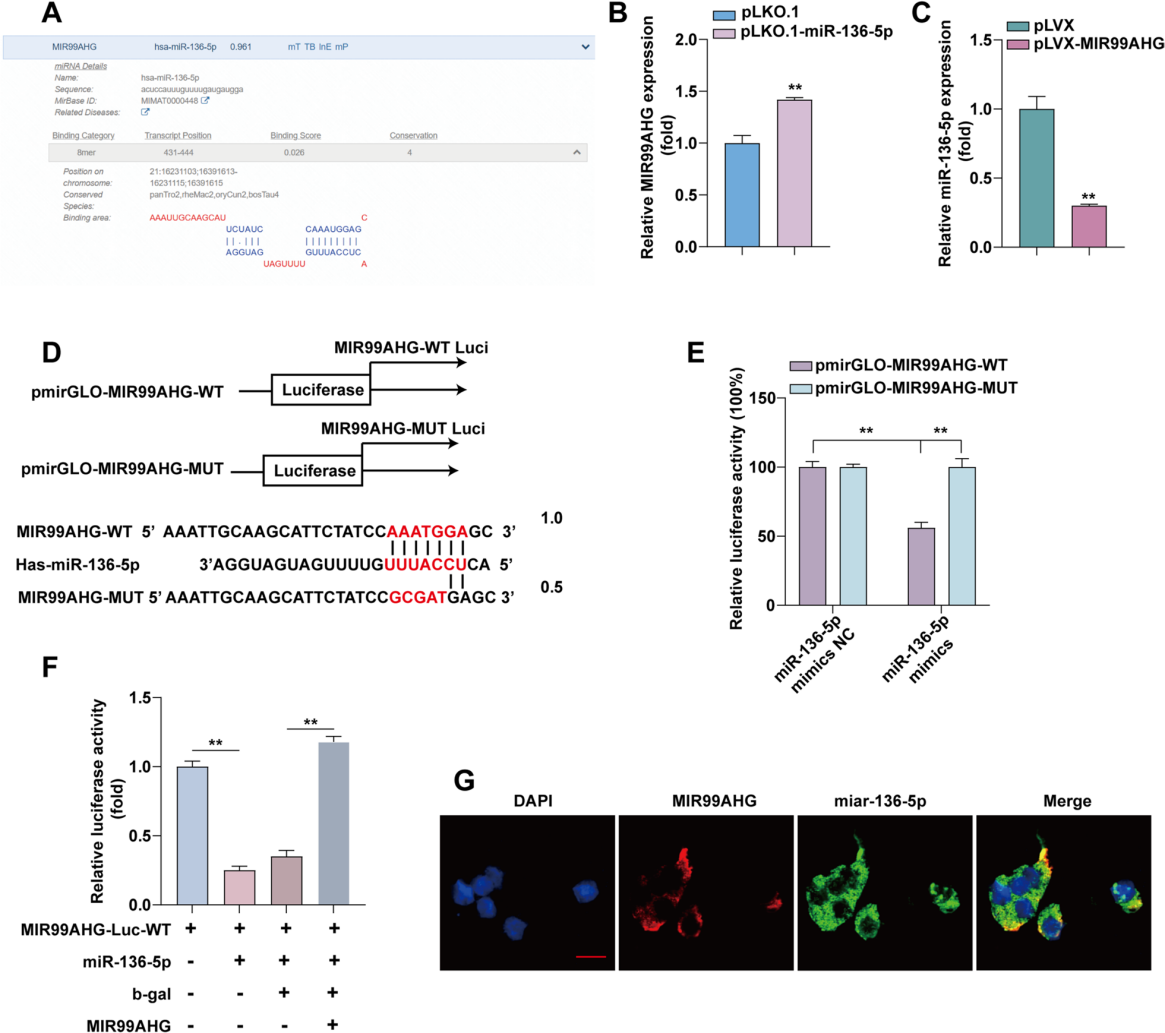
MIR99AHG targets miR-136-5p to exert its tumor suppressive effects in LUAD.** A** DIANA database analysis of LncRNA MIR99AHG binding sites to miR-136-5p. **B** Expression of MIR99AHG was examined by RT-qPCR after knocking down miR-136-5p. **C** Expression of miR-136-5p was examined by RT-qPCR after overexpression of MIR99AHG. **D** Putative binding sequence between MIR99AHG and miR-136-5p. **E** After transfection in HEK293T cells according to experimental subgroups, luciferase activity analysis (n = 3). **F** Luciferase reporters containing MIR99AHG-Luc-WT were co-transfected with or without miR-136-5p and MIR99AHG, Luciferase activity was determined using a dual luciferase reporter system. **G** FISH results show the colocalization of MIR99AHG and miR-136-5p in the cytoplasm of LUAD cells

Analysis of the DIANA database (https://diana.e-ce.uth.gr/lncbasev3) revealed that MIR99AHG has miR-136-5p binding sites (Fig. 2A). In A549 cells, stable knockdown of miR-136-5p resulted in upregulation of MIR99AHG (Fig. 2B). Stable overexpression of MIR99AHG resulted in miR-136-5p downregulation (Fig. 2C). These results suggest that MIR99AHG and miR-136-5p are competitively expressed. To further determine the interaction between miR-136-5p and MIR99AHG, we constructed luciferase vectors for wild-type (WT) and mutant (MUT) MIR99AHG (Fig. 2D). Using a dual luciferase reporter gene assay, we found that the 293T cells transfected with MIR99AHG-WT plasmid and miR-136-5p mimics, but not MIR99AHG-WUT plasmid, showed significantly reduced luciferase activity (Fig. 2E). After transfection of the MIR99AHG expression plasmid, the decreased luciferase activity after transfection of miR-136-5p was restored (Fig. 2F). FISH analysis reveals co-localization of MIR99AHG and miR-136-5p in the cytoplasm of LUAD cells (Fig. 2G).

### MiR-136-5p promotes EMT and fibrosis in LUAD

**Fig. 3:**
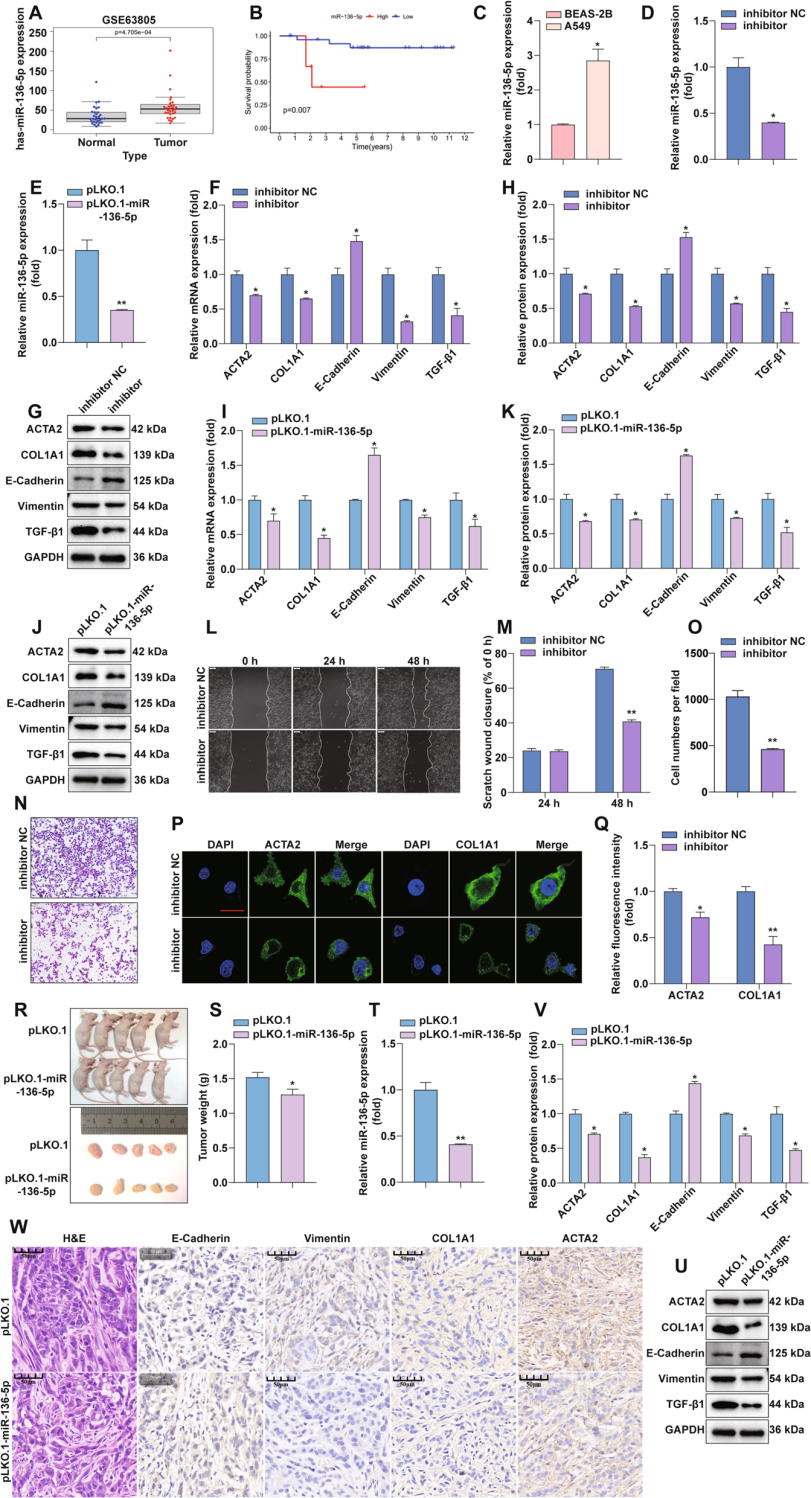
MiR-136-5p promotes EMT and fibrosis in LUAD.** A** GEO database analysis-miR-136-5p expression differences in LUAD and normal lung tissues. **B** Analysis of miR-136-5p expression in relation to survival of LUAD using GEO database. **C** RT-qPCR detection of miR-136-5p expression differences in BEAS-2B and A549. **D** MiR-136-5p inhibitor treatment A549 after RT-qPCR to detect the mRNA expression of miR-136-5p. **E** Stable knockdown of miR-136-5p and RT-qPCR to detect the knockdown efficiency of miR-136-5p. **F** MiR-136-5p inhibitor treatment of A549 after RT-qPCR to detect the expression of EMT and fibrosis markers. **G**, **H** MiR-136- 5p inhibitor treatment of A549, WB to detect the protein expression of TGF-β1, E-Cadherin, Vimentin, ACTA2 and COL1A1. Statistical quantification by ImageJ software. **I** Stable knockdown of miR-136-5p followed by RT-qPCR to detect the expression of EMT and fibrosis markers. **J**, **K** After stably knocking out miR-136-5p, the expression of related proteins was detected by WB and statistically quantified by ImageJ software. **L**, **M** Wound scratch assay was performed to monitor cell migration after MiR-136-5p inhibitor treatment of A549 cells, Statistical data are shown. **N**, **O** Invasion assay was performed to determine cell invasion after miR-136-5p inhibition treatment of A549 cells. **P**, **Q** MiR-136-5p inhibitor A549 was followed by an Immunofluorescence assay performed to detect the expression of the fibrosis-associated protein. Statistical data are shown. **R** Subcutaneous tumorigenic demonstration in nude mice. **S** Tumor weights were represented as the means of tumor weights ± SD. **T** RT-qPCR to confirm the effect of miR-136-5p knockdown in tumor tissues. **U**, **V** WB to detect the expression of miR-136-5p stable knockdown of TGF-β1, E-Cadherin, Vimentin, ACTA2 and COL1A1 in tumor tissues. Statistical data are shown. **W** The tumor sections underwent IHC staining and H&E staining

We analyzed the GEO dataset and found that miR-136-5p expression in LUAD was significantly higher than in normal lung tissue (Fig. 3A). The correlation of miR-136-5p expression with OS in LUAD patients was analyzed using the UALCAN database (http://ualcan.path.uab.edu/analysis.html), where patients were dichotomized using median miR-136-5p expression. We observed that lower miR-136-5p expression was associated with worse OS (Fig. 3B). The miRNA was extracted from BEAS-2B and A549 cells and subjected to RT-qPCR, and the results show that MiR-136-5p expression was significantly higher in A549 than in BEAS-2B (Fig. 3C). After transient transfection of inhibitor of miR-136-5p (a single-stranded RNA molecule that inhibits the function of miR-136-5p) and stable knockdown of miR-136-5p in A549 cells (Fig. 3D and E), the RNA and protein expression of E-Cadherin was elevated. Protein expression of TGF-β1, Vimentin, ACTA2, and COL1A1 was decreased after inhibitor treatment (Fig. 3F–H). Similar results were obtained in stable knockdown miR-136-5p cells (Fig. 3I–K). To verify the effect of miRNA-136-5p on EMT and fibrosis, the inhibitory effect of miR-136-5p inhibition on A549 cell invasion was confirmed by wound scratch and invasion assays (Fig. 3L–O). Immunofluorescence assay confirmed that inhibiting miR-136-5p could lead to the lower expression of ACTA2 and COL1A1 proteins in A549 cells (Fig. 3P). And we quantified the immunofluorescence results (Fig. 3Q). The above results suggest that miR-136-5p can promote EMT and fibrosis.

To determine the effect of miR-136-5p on LUAD growth in vivo, A549 cells with a lentiviral vector producing shRNA against miR-136-5p were inoculated into the dorsal region of the mouse, and lentivector-infected A549 cells carrying a control shRNA were inoculated into the dorsal region of the same mouse as a control. When the tumors grew to the appropriate size, the complete tumor blocks were removed and weighed. The results are shown in Fig. 3R, S. The tumors from the miR-136-5p stable knockdown cell group were smaller in size and weighed less than the pLKO.1 control group. The expression of miR-136-5p in tumor tissues was examined by RT-qPCR, and it was determined that miR-136-5p remained stably suppressed in nude mice (Fig. 3T). WB detected the expression levels of TGF-β1-induced EMT and fibrotic protein in tumor tissues stably downregulated by miR-136-5p (Fig. 3U, V). We performed histopathological analysis of nude mouse tumors using H&E staining, and the expression levels of related proteins in tumor of nude mice were detected by IHC. As shown in Fig. 3W, the nuclei of the miR-136-5p knockdown group had obvious wrinkling and unclear cell contours; the expression of E-Cadherin, an EMT-related protein, increased, and the expression of Vimentin, ACTA2, and COL1A1, fibrosis markers, decreased. These results suggest that the knockdown of miR-136-5P inhibited the expression of EMT and fibrosis markers in LUAD.

### USP4 is a direct target of miR-136-5p

**Fig. 4:**
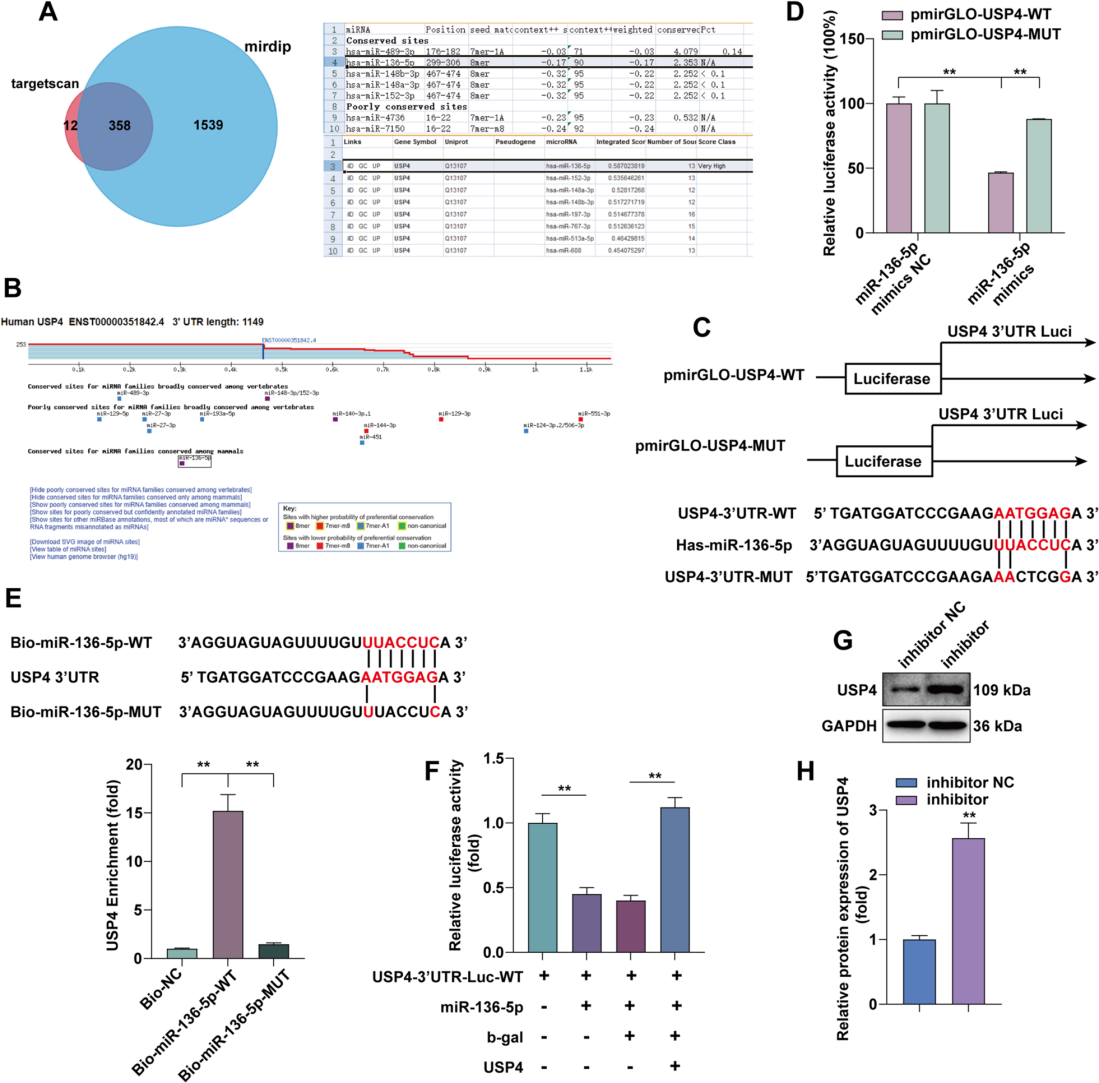
USP4 is a direct target gene of miR-136-5p. **A**, **B** TargetScan and mirDIP prediction of the target gene of miR-136-5p. Funrich made a Wayne diagram. **C** WT and MUT of the 3’UTR of USP4 luciferase reporter plasmids are shown schematically. **D** Luciferase activity analysis in HEK293T cells after transfection according to experimental subgroups (n = 3). **E** Enrichment of USP4 in A549 cells transfected with miR-136-5p-WT and miR-136-5p-MUT plasmids. **F** Luciferase reporters containing USP4-Luc-WT were co-transfected with or without miR-136-5p, and USP4, Luciferase activity was determined using a dual luciferase reporter system. **G**,** H** After treatment of A549 with miR-136-5p inhibitor, the protein level of USP4 was detected by WB and quantitatively analyzed by ImageJ.

To explore the molecular mechanism of the effect of miR-136-5p on lung fibrosis, we predicted the target genes of miR-136-5p based on the TargetScan (http://www.targetscan.org/vert_72/) and mirDIP database (http://ophid.utoronto.ca/mirDIP/index.jsp). We took the predicted target genes from both databases to take the intersection by FunRich software. MiR-136-5p has 358 common target genes in TargetScan and mirDIP database. Among them, USP4 is the miR-136-5p target gene, as provided in Fig. 4A and B. MiR-136-5p in Targetscan can target the 3′UTR of USP4. To further determine the interaction between miR-136-5p and USP4, we constructed luciferase vectors for WT and MUT USP4 (Fig. 4C). Using a dual luciferase reporter gene assay, we found that 293T cells transfected with USP4-WT and miR-136-5p mimics, instead of USP4-WUT, showed significantly reduced luciferase activity (Fig. 4D). Further biotin-labeled miRNA pull-down assay demonstrated that it was miR-136-5p-WT, instead of miR-136-5p-MUT, that enriched USP4 (Fig. 4E). After transfection of USP4 expression plasmid, the decreased luciferase activity after transfection of miR-136-5p was restored (Fig. 4F). Next, we transfected miR-136-5p inhibitor in A549 cells and found that the protein expression of USP4 was upregulated (Fig. 4G, H). The above results demonstrate that USP4 is an important target of miR-136-5p in LUAD cells, and miR-136-5p can effectively regulate the expression of USP4.

### USP4 deubiquitination maintains ACE2 stability and thus inhibits EMT and fibrosis in LUAD

**Fig. 5:**
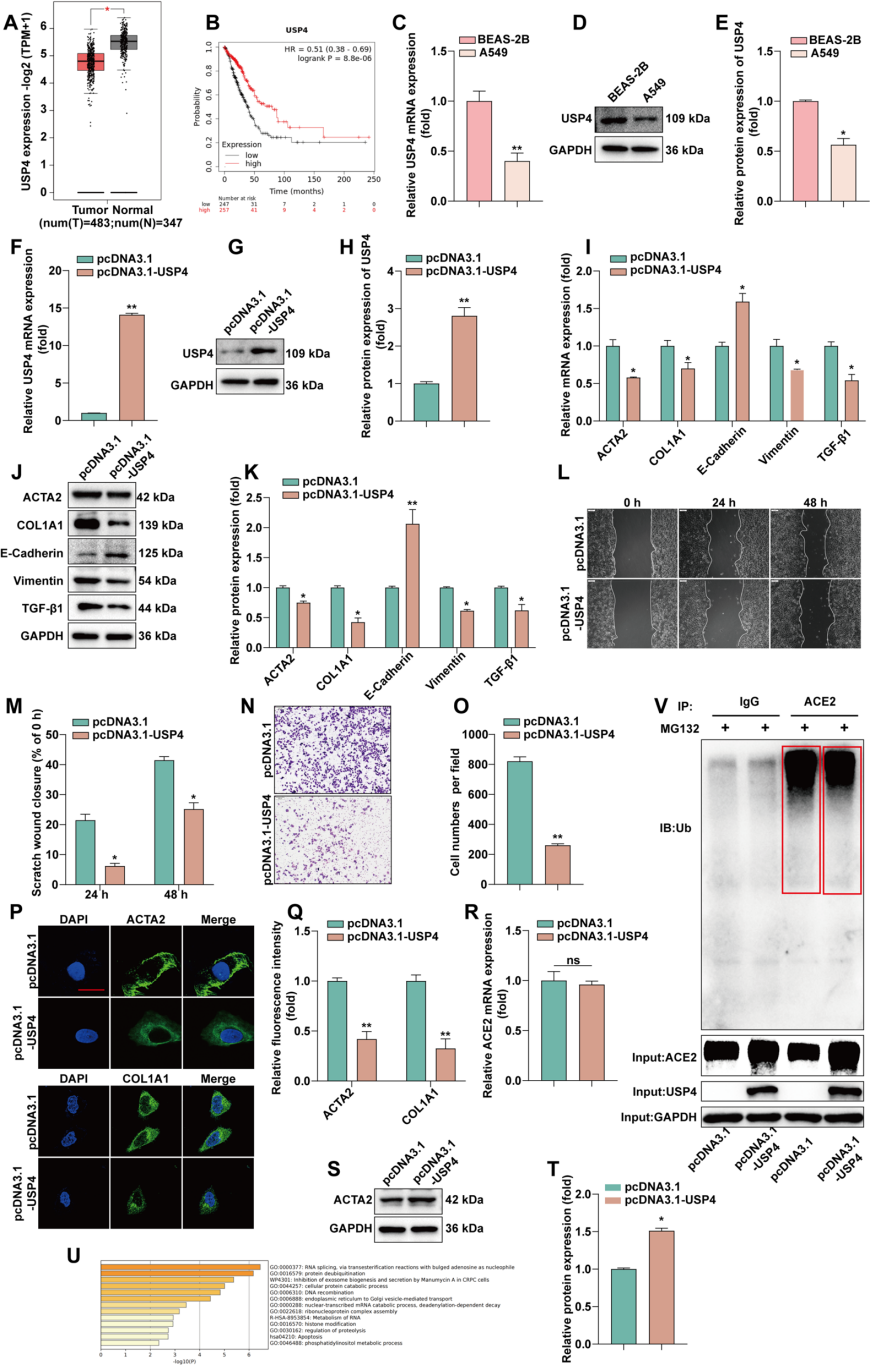
USP4 deubiquitination maintains ACE2 stability and thus inhibits EMT and fibrosis in LUAD.** A** Analysis of USP4 expression in LUAD and normal lung tissues using the GEPIA database. **B** Analysis of USP4 expression in relation to OS of LUAD patients using Kaplan–Meier plotter. **C** Detection of USP4 mRNA expression in BEAS-2B and A549 by RT-qPCR. **D**, **E** Detection of USP4 protein expression in BEAS-2B and A549 by WB and statistical quantification by ImageJ software. **F** RT-qPCR detection of USP4 mRNA expression levels after overexpression of USP4. **G**,** H** WB detection of USP4 protein expression levels after overexpression of USP4 and statistical quantification by ImageJ software. **I** Overexpression of USP4 followed by RT-qPCR for EMT and fibrosis marker expression. **J**, **K** The protein expressions of TGF-β1, E-cadherin, vimentin, ACTA2 and COL1A1 were detected by WB after overexpression of USP4. Statistical quantification by ImageJ software. **L**, **M** After overexpression of USP4, cell migration was monitored by wound scratch assay. **N**, **O** After the expression of USP4, cell invasion was determined by invasion assay. And counted statistically by ImageJ. **P**, **Q** After overexpression of USP4, an immunofluorescence assay was performed to detect the expression of fibrosis-associated proteins. Statistical data are shown. **R** Detection of ACE2 mRNA expression by RT-qPCR after overexpression of USP4. **S**, **T** Detection of ACE2 protein expression by WB after overexpression of USP4 and quantified by ImageJ software statistics. **U** Functional enrichment analysis of USP4 by Metascap website. **V** Analysis of ubiquitinated degradation of ACE2 after overexpression of USP4 by Co-IP assay

To verify the correlation between USP4 and fibrosis, we analyzed the expression of USP4 in LUAD and normal lung tissues through the GEPIA database. The results (Fig. 5A) show that USP4 expression in LUAD is significantly lower than that in normal lung tissues. The Kaplan–Meier curve (Fig. 5B) shows that higher expression of USP4 is associated better OS of patients. We next examined USP4 expression at the cellular level by RT-qPCR and WB. The results show that USP4 expression was lower in A549 cells compared with BEAS-2B (Fig. 5C–E). To verify the effect of USP4 on fibrosis, USP4 was overexpressed in A549 (Fig. 5F–H). The expression of TGF-β1-induced EMT and fibrosis related proteins was detected by RT-qPCR and WB after overexpression of USP4 (Fig. 5I–K). Further, the inhibitory effect of USP4 overexpression on A549 cell invasion was confirmed by wound scratch and invasion assays (Fig. 5L–O). USP4 was confirmed to inhibit the expression of ACTA2 and COL1A1 proteins in A549 cells by immunofluorescence assay (Fig. 5P and Q).

To find the correlation between USP4 and fibrosis, we found that ACE2 plays an important role in the process of lung fibrosis and EMT. It has been reported that ACE2 can be modified by MDM2 ubiquitination [24], while USP4 belongs to a member of the deubiquitin-specific peptidase family. We examined the mRNA expression and protein expression of ACE2 after overexpression of USP4. The overexpression of USP4 did not change the RNA level of ACE2 compared to the control group (Fig. 5R). Still, the protein level increased compared to the control group (Fig. 5S, T). USP4 has deubiquitinating kinase properties and can regulate the process of substrate deubiquitination. Functional enrichment analysis of USP4, according to the Metascap website, revealed that USP4 is involved in several cellular signaling pathways, including protein deubiquitination (Fig. 5U). To verify whether USP4 may regulate ACE2 expression through deubiquitination, we performed Co-IP experiments, which showed that USP4 could interact with ACE2 and that overexpression of USP4 upregulated ACE2 expression (Fig. 5V). These results suggest that USP4 can maintain ACE2 protein stability through deubiquitination, which in turn inhibits EMT and fibrosis in LUAD.

### ACE2 is involved in EMT and fibrosis in LUAD

**Fig. 6:**
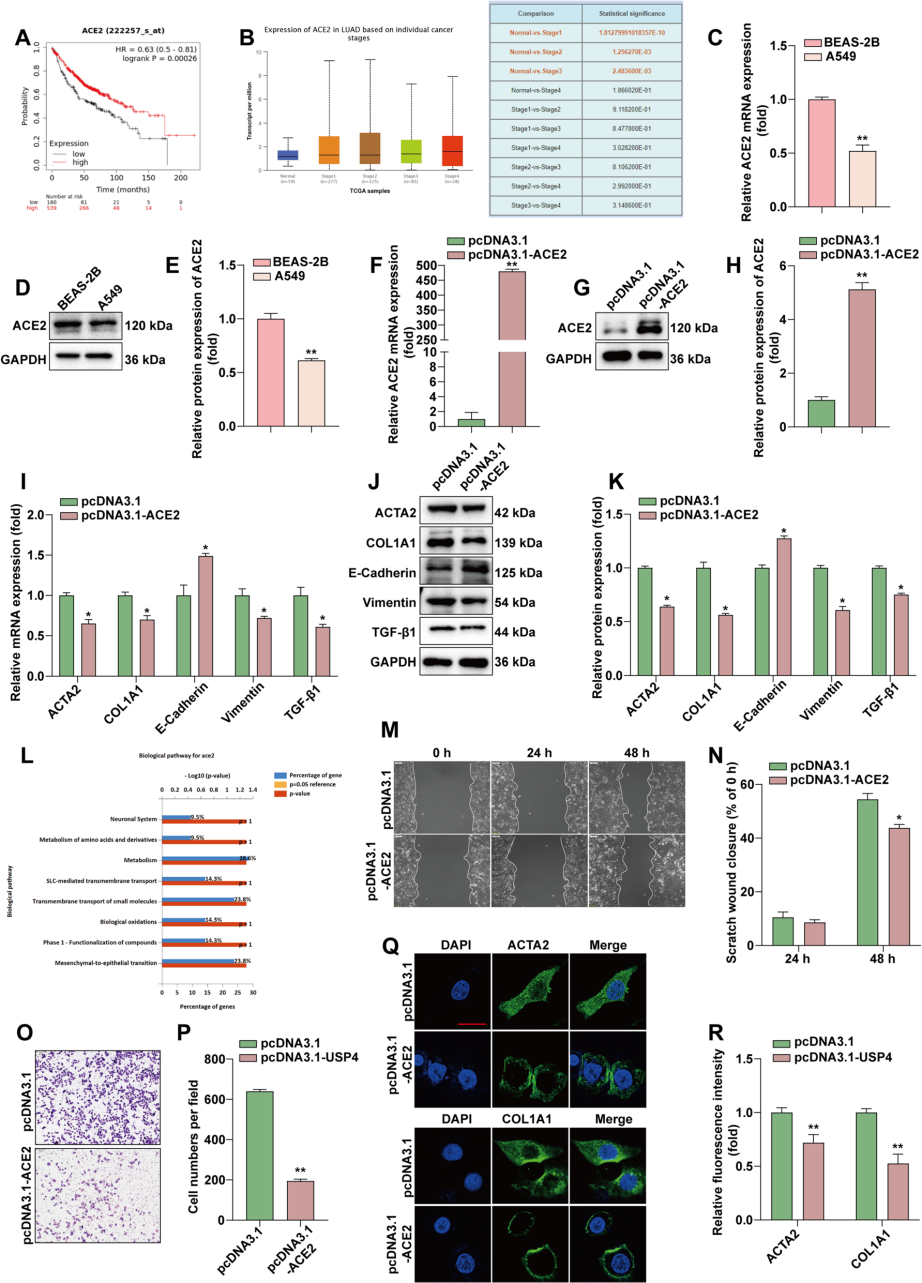
Overexpression of ACE2 is involved in EMT and fibrosis in LUAD.** A** Kaplan–Meier plotter analysis of ACE2 expression in relation to survival in lung cancer patients. **B** Higher ACE2 levels were positively correlated with cancer stage. **C** RT-qPCR detection of ACE2 in BEAS-2B and A549. **D**,** E** WB detection of ACE2 protein expression in BEAS-2B and A549, and statistical quantification of ACE2 protein expression by ImageJ software. **F** After overexpression of ACE2, RT-qPCR detection of ACE2 mRNA expression levels. **G**,** H** After overexpression of ACE2, WB to detect ACE2 protein expression levels after overexpression of ACE2. ImageJ software for statistical quantification. **I** RT-qPCR to detect EMT and fibrosis marker expression after overexpression of ACE2. **J**, **K** WB to detect the protein expressions of TGF-β1, E-cadherin, vimentin, ACTA2 and COL1A1 after overexpression of ACE2. ImageJ software for statistical quantification of protein expression. **L** Functional enrichment of ACE2 analysis. **M**, **N** Cell migration was monitored by wound scratch assay after overexpression of ACE2. **O**, **P** Cell invasion was measured by invasion assay after overexpression of ACE2 and counted statistically by ImageJ. **Q**, **R** Immunofluorescence assay to detect fibrosis-associated protein expression after overexpression of ACE2. Statistical data are shown

By analyzing clinic pathological factors, we found that ACE2 was associated with poor prognosis in patients. Decreased ACE2 levels predicted a poorer OS in patients (*P* = 0.00026, Fig. 6A). Higher ACE2 levels were positively correlated with the cancer stage (*P* < 0.01, Fig. 6B). ACE2 expression was detected by RT-qPCR and WB (Fig. 6C–E), Lower ACE2 expression in A549 cells compared with BEAS-2B. ACE2 was next overexpressed in A549 cells (Fig. 6F–H). RT-qPCR and WB examined the expression of related proteins after overexpression of ACE2 (Fig. 6I–K). Overexpression of ACE2 increased E-Cadherin expression and decreased the expression of TGF-β1, Vimentin, ACTA2, and COL1A1. Functional enrichment analysis also indicated that ACE2 is involved in mesenchymal-to-epithelial transition (MET) (Fig. 6L). The mutual transformation between epithelialization and mesenchymalization of tumor cells is closely related to tumor recurrence and distant metastasis. EMT and MET jointly participate in the process of tumor development. To verify the effect of ACE2 on EMT, we performed a cell scratch assay and invasion assay to detect cell migration. The scratch assay ability of cells in the ACE2 overexpression group in the scratch assay was significantly lower than that of the control group (Fig. 6M, N). The invasion assay demonstrated that the migratory ability of A549 cells was significantly reduced after overexpression of ACE2 (Fig. 6O and P). To verify the effect of ACE2 on fibrosis, we performed immunofluorescence experiments, as shown in Fig. 6Q and R, overexpression of ACE2 inhibited the expression of ACAT2 and COL1A1. The above results indicate that ACE2 overexpression can inhibit EMT and fibrosis in LUAD.

### MIR99AHG inhibits pulmonary fibrosis in rats

**Fig. 7:**
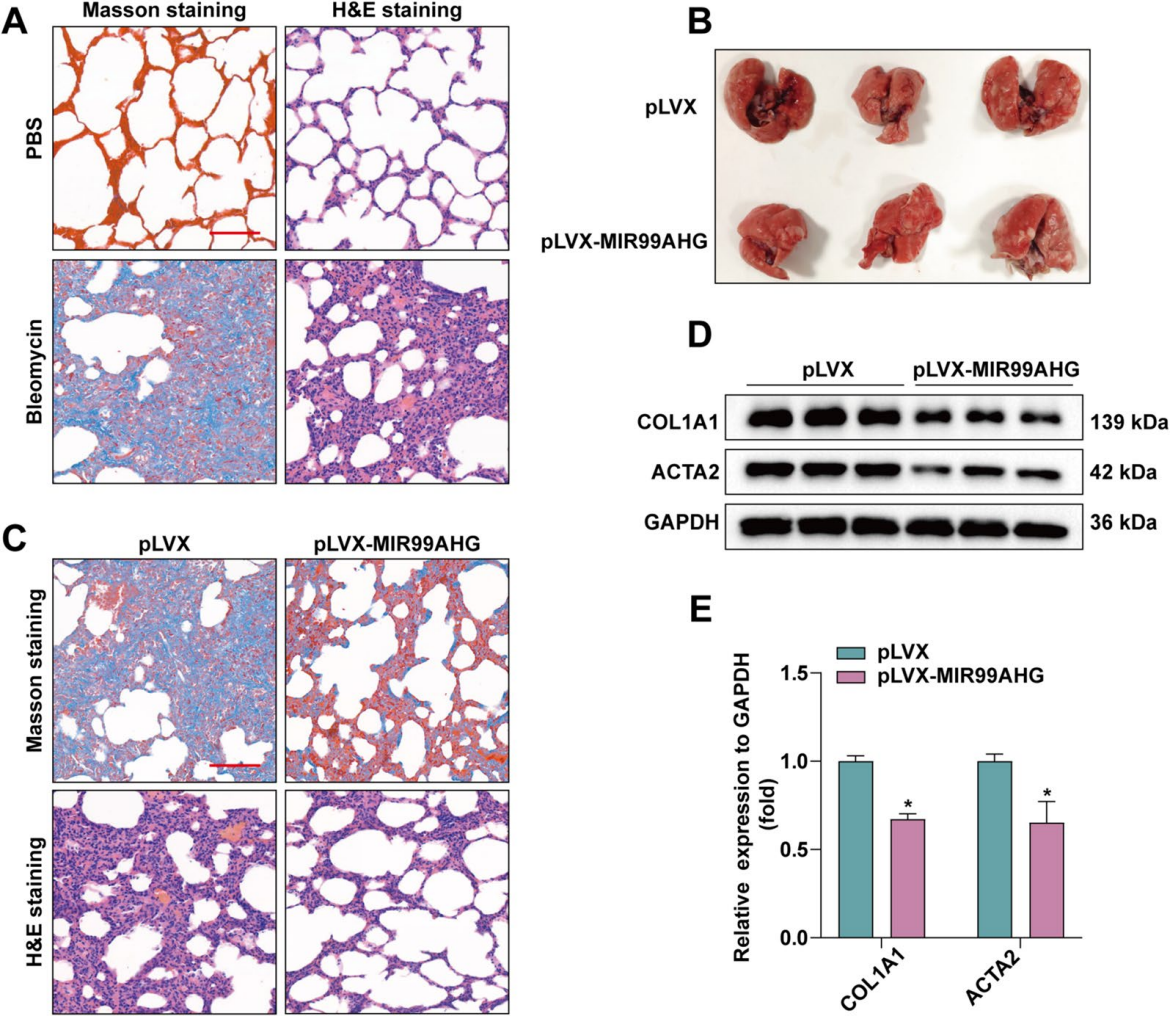
MIR99AHG inhibited lung fibers in rats.** A** Masson staining and H&E staining of lung tissues of SD rats after 21 days of bleomycin administration. **B** Lung tissue of SD rats after lentivirus injection. **C** Masson staining and H&E staining of lung tissues of SD rats after injection of lentivirus overexpressing MIR99AHG or control PLVX. **D **and **E** WB to detect ACTA2 and COL1A1 protein expression levels in lung tissues. ImageJ software was used for statistical quantification

The SD rat model of bleomycin-induced pulmonary fibrosis is widely used to explore the pathogenesis and potential new therapies of pulmonary fibrosis. We used this SD rat model to investigate whether overexpression of MIR99AHG in vivo has therapeutic benefits. After 21 days of bleomycin administration to SD rats, three rats were randomly selected to take their lung tissues for H&E staining and Masson staining and the results showed that we successfully constructed a rat pulmonary fibrosis model (Fig. 7A). To determine the potential role of MIR99AHG in the development of pulmonary fibrosis using bleomycin administered to SD rats for 28 days, we treated the rats with an intraperitoneal injection of either MIR99AHG-overexpressing lentivirus or a control PLVX lentivirus. The lung tissue of SD rats was taken after 30 days (Fig. 7B). As shown in Fig. 7C, Masson staining showed that compared with the control group, the blue staining of the lung tissue of the rats treated in the experimental group decreased. H&E staining showed that compared with the control group, the lung tissue structure of the experimental group was clear. Finally, WB was used to further detect and qualitatify the protein levels of ACTA2 and COL1A1 in SD rats lung tissue (Fig. 7D and E). The results showed that the levels of ACTA2 and COL1A1 were reduced in the pLVX-MIR99AHG group. Altogether, our results suggest that MIR99AHG can inhibit the progression of pulmonary fibrosis in vivo.

## Discussion

LncRNAs have been shown to play a key role in developing several diseases [25]. Pulmonary fibrosis is a respiratory disease caused by interstitial lung lesions, and a large body of data supports the association of pulmonary fibrosis with lung cancer [26], which is an independent risk factor for lung cancer, and lung cancer frequently occurs in patients with long-term chronic pulmonary fibrosis [27]. LncRNAs have been shown to play a role in fibrosis. In the present study, we found that lncRNA MIR99AHG expression was downregulation in LUAD compared to the corresponding non-tumor tissues. Furthermore, downregulation of MIR99AHG in LUAD tissues was associated with poor prognosis and may be an independent prognostic indicator. These results suggest that MIR99AHG may have an important role in the progression of pulmonary fibers in LUAD.

MiRNAs are a small class of noncoding RNAs involved in many fundamental cellular processes, including extracellular matrix remodeling, alveolar epithelial cell apoptosis, and EMT [28, 29]. Overexpression of MIR99AHG has been reported to increase the expression of the miR-99a/let-7c/miR-125b2 cluster, synergistically acting as a tumor suppressor gene [13]. In addition, MIR99AHG can also act as a ceRNA to antagonize other miRNAs [30]. MiRNA sequestration is the most frequently reported mechanism by which lncRNAs exert their regulatory functions. We found that miR-136-5p can bind MIR99AHG through the LncBase database and verified the binding between MIR99AHG and miR-136-5p using dual luciferase reporter gene and FISH assays. Further functional experiments showed that MIR99AHG absorbed miR-136-5p to inhibit LUAD fibrosis progression. To investigate the target genes of the MIR99AHG/miR-136-5p pathway in LUAD, we combined two databases to identify USP4 as a downstream effector of the MIR99AHG/miR-136-5p axis. The role of USP4 in many pathological and physiological processes has been reported; USP4 promotes breast cancer invasive migration through the relaxin2-mediated TGF-β1/Smad2/MMP-9 pathway [31]; USP4 inhibits myogenic cell differentiation by catalyzing MyoD activity [32]; The H19/miR-148a/USP4 axis promotes hepatic stellate cells by enhancing and TGF-β signaling in hepatocytes to promote liver fibrosis [33]. Our results suggest that MIR99AHG inhibits pulmonary fibrosis through the miR-136-5p/USP4 pathway. However, only a few studies have focused on the involvement of lncRNA in lung fibrosis progression. Our data provide new insights into the role of MIR99AHG in regulating the formation of pulmonary fibrosis. This finding may provide a checkpoint target for tissue fibrosis and fibrosis in patients with cancer.

Reports show that the ACE2/Ang(1–7)/Mas receptor pathway antagonizes the ACE/AngII/AT1R axis and that the ACE2/Ang-(1–7)/Mas axis protects against pulmonary fibrosis by inhibiting the MAPK/NF-κB pathway [23, 34]; AngII upregulates the expression of the pro-fibrotic cytokine TGF-β1, which is involved in both the fibroblast to myofibroblast conversion and collagen accumulation [35], and ACE2 breaks down AngII into Ang-(1–7) and inhibits fibrosis development [36]. In this paper, overexpression of ACE2 inhibits the expression of EMT and fibrosis markers. Recent studies have found that ACE2 can be ubiquitinated by MDM2 [24], and USP4 is a deubiquitinating enzyme that excises protein substrates and links them to ubiquitin, reversing the ubiquitination degradation process, and maintains cells stability growth [37], and overexpression of USP4 can promote colorectal cancer growth by deubiquitinating the oncogene PRL-3 [38]. In this paper, we showed that USP4 could deubiquitinate ACE2 to maintain the protein stability of ACE2 and thus inhibit the expression of EMT and fibrosis markers. This result was confirmed by scratch assays and immunofluorescence. However, the specific sites of action of USP4 on ACE2 deubiquitination and whether other members of the USP family can maintain ACE2 stability by deubiquitination still need to be further explored and validated.

**Fig. 8:**
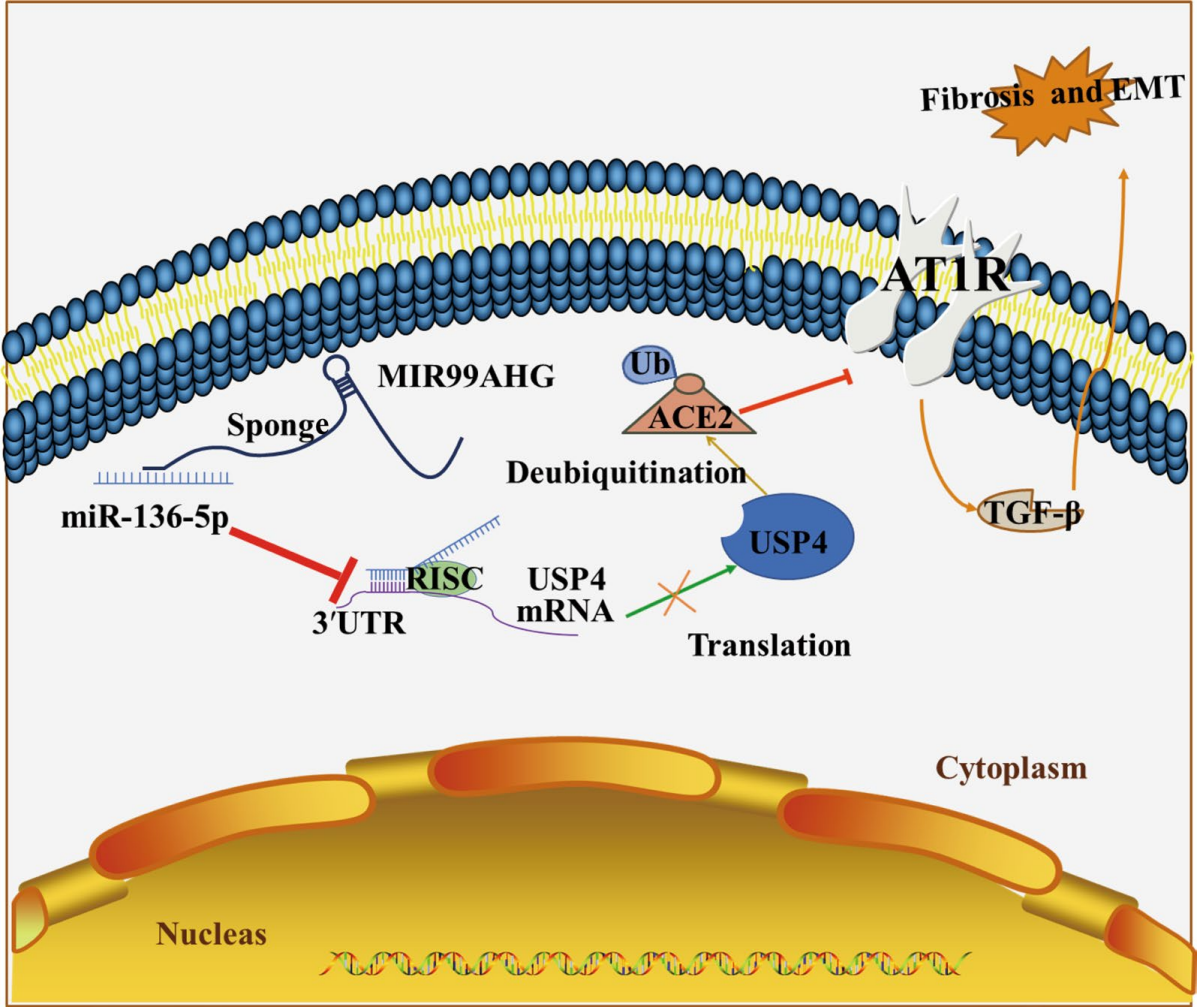
Graphical summary of the regulatory mechanisms of MIR99AHG in the progression of pulmonary fibrosis. MIR99AHG acted as a sponge of miR-136-5p to inhibit miR-136-5p expression, upregulated the expression of USP4, and enhanced the stability of ACE2 protein, thereby inhibiting EMT in pulmonary fibrosis

In the present study, we investigated the role and mechanism of MIR99AHG in LUAD. We report that MIR99AHG is preferentially localized in the cytoplasm and that MIR99AHG acts as ceRNA to increase the expression of USP4 mRNA by competing with miR-136-5p to maintain ACE2 protein stability. ACE2 is widely expressed in cardiomyocytes, cardiac fibroblasts and coronary endothelial cells, cleaves AngII to Ang-(1–7) polypeptide, which has cardioprotective, vasodilatory, anti-growth and anti-proliferative effects. Attenuates pulmonary fibrosis by regulating the ACE2/Ang-(1–7)/Mas axis. In A549 cells, MIR99AHG and ACE2 levels were always parallel, and the reduced levels inhibited EMT and thus the progression of pulmonary fibrosis. The regulatory mechanism of MIR99AHG is shown in Fig. 8.

## Conclusion

In summary, we comprehensively investigated the functional role and molecular mechanisms of MIR99AHG in LUAD. Our findings suggest that MIR99AHG expression is upregulated in A549 cells and LUAD tissues. High MIR99AHG expression levels were an independent prognostic factor for the overall survival of LUAD patients. Our study suggests that the MIR99AHG/miR-136-5p/USP4/ACE2 signaling axis regulates the process of EMT and lung fibrosis. Our findings suggest that MIR99AHG plays a key role in the progression of pulmonary fibrosis and is a potentially effective target for pulmonary fibrosis therapy.

## Supplementary Information

**Additional file 1: Table S1.** Plasmid construction primer sequences. **Table S2.** Primers of qRT-PCR.

**Additional file 2: Figure S1.** Abnormal expression of MIR99AHG in LUAD tissues. **A**. H&E staining of the LUAD tumor tissues and non-tumor tissues. **B**. Masson staining of tissue from LUAD with fibrosis. **C**. The expression levels of MIR99AHG in 20 paired LUAD tissues and non-tumor specimens were determined using RT-qPCR. **D, E**. FISH was used to determine the expression of MIR99AHG in LUAD tissue and the paired non-tumor sample. Statistical data are shown. **F**. FISH was performed to determine the subcellular distribution of MIR99AHG in A549 cells. ***P < 0.001.

## Data Availability

The datasets used and/or analyzed during the present study are available from the corresponding author on reasonable request.

## Abbreviations

LncRNAs: Long non-coding RNAs lncRNAs
LUAD: Lung adenocarcinoma
FISH: Fluorescence in situ hybridization
ACE2: Angiotensin converting enzyme 2
USP4: Ubiquitin specific protease 4
EMT: Epithelial mesenchymal transition
ceRNA: Competitive endogenous RNA
FBS: Fetal bovine serum
WB: Western Blot
IHC: Immunohistochemistry
Co-IP: Co-immunoprecipitation
RT-qPCR: Quantitative reverse transcription PCR
SDS-PAGE: Sodium dodecyl sulphate-polyacrylamide gel electrophoresis
WT: Wild type
MUT: Mutant
