# Supplementary material for: MIR99AHG inhibits EMT in pulmonary fibrosis via the miR-136-5p/USP4/ACE2 axis
Source: J Transl Med. 2022 Sep 23;20:426. doi: 10.1186/s12967-022-03633-y (PMC9502606; doi:10.1186/s12967-022-03633-y)
Supplement: Supplementary file 1 — Additional file 1: Table S1. Plasmid construction primer sequences. Table S2. Primers of qRT-PCR. [file 12967_2022_3633_MOESM1_ESM.docx]

**Additional Tables**

**Table S1**

| Plasmid name | sequences（5' - 3'） |
| --- | --- |
| pcDNA3.1-ACE2 | F: GGGAGACCCAAGCTGGCTAGCATGTCAAGCT  CTTCCTGGCT |
|  | R: AGCTTAAGTTTAAACGCTAGCCTAAAAGGA  GGTCTGAACATCATC |
| pcDNA3.1-USP4 | F: GGGAGACCCAAGCTGGCTAGCATGGCGGA  AGGTGGAGGCT |
|  | R: AGCTTAAGTTTAAACGCTAGCTTAGTTGGTG  TCCATGCTGCAAG |
| pmirGLO-USP4-  WT-3′UTR- Luci | F: TGTTTAAACGAGCTCGCTAGCTGCTGACTC  CACGATCCTGCCA |
|  | R: GACTCTAGACTCGAGGCTAGCACCCCCCCT  TTGGCGAGTC |
| pmirGLO-USP4-  MUT-3′UTR- Luci | F: TCCCGAAGAACTCGGAGGAACACAG  GCGCTGAGTATGGAGCAG |
|  | R: TGTGTTCCTCTTCTTCGGGATCCATCAGACA  TACTCCATTGAGT |
| pmirGLO-MIR99AHG-WT-3′UTR- Luci | F: TGTTTAAACGAGCTCGCTAGCGGCCACTGTC  TGCAGCA |
|  | R: GACTCTAGACTCGAGGCTAGCCTGTGTCATG  GAGGACCAATTTA |
| pmirGLO-MIR99AHG-MUT-3′UTR- Luci | F: CATTCTATCC*G*CGATGAGCTCTTTCTGAGATG  AAGAGAATTCTCAATGT |
|  | R: AGAAAGAGCTCATCGCGGATAGAATGCTTGC  AATTTATTTGGATAATTATC |
| pLKO.1-EGFP-  miR-136-5P | ACCGGCGGCGCTAGGATCATCAACTCCATCATCAAAATCTACAAATGGAGTCAAGTATTCTGGTCACAGAATACAACTCCATCATCAAAATCTACAAATGGAGTCAAGATGATCCTAGCGCCGCC TTTTTTGAATT |

Table S1: Plasmid construction primer sequences

**Table S2**

| Primer name | | sequences（5'-3'） |
| --- | --- | --- |
| GAPDH | F: GGAGCGAGATCCCTCCAAAAT | |
|  | R: GGCTGTTGTCATACTTCTCATGG | |
| ACE2 | F: TCATGCCTATGTGAGGGCAA | |
|  | R: ACTTCTCGGCCTCCTTGAAT | |
| USP4 | F: CCCTACCGAGGCGTGGAATA | |
|  | R: CGACTTTGCAGTGCTTGACA | |
| E-Cadherin | F: CGAGAGCTACACGTTCACGG | |
|  | R: GGGTGTCGAGGGAAAAATAGG | |
| Vimentin | F: GACGCCATCAACACCGAGTT | |
|  | R: CTTTGTCGTTGGTTAGCTGGT | |
| ACTA2 | F: AGCGTGGCTATTCCTTCGTT | |
|  | R: GCCCATCAGGCAACTCGTAA | |
| COL1A1 | F: GAGACGTGTGGAAACCCGAG | |
|  | R: GTGGTTTCTTGGTCGGTGGG | |
| miR-136-5p | F: CGGCACTCCATTTGTTTTGAT | |
|  | R: ACTGCAGGGTCCGAGGTATT | |
|  | RT:GTCGTATCGACTGCAGGGTCCGAGGTATTCGCAGTCGATACGACTCCAT | |
| U6 | F: CTCGCTTCGGCAGCACA | |
|  | R: AACGCTTCACGAATTTGCGT | |
|  | RT: AACGCTTCACGAATTTGCGT | |
| MIR99AHG | F: GGCCACTGTCTGCAGCA | |
|  | R: CTGTGTCATGGAGGACCAATTTA | |

Table S2: Primers of qRT-PCR
