# Supplementary material for: MIR99AHG inhibits EMT in pulmonary fibrosis via the miR-136-5p/USP4/ACE2 axis
Source: J Transl Med. 2022 Sep 23;20:426. doi: 10.1186/s12967-022-03633-y (PMC9502606; doi:10.1186/s12967-022-03633-y)
Supplement: Supplementary file 2 — Additional file 2: Figure S1. Abnormal expression of MIR99AHG in LUAD tissues. A. H&E staining of the LUAD tumor tissues and non-tumor tissues. B. Masson staining of tissue from LUAD with fibrosis. C. The expression levels of MIR99AHG in 20 paired LUAD tissues and non-tumor specimens were determined using RT-qPCR. D, E. FISH was used to determine the expression of MIR99AHG in LUAD tissue and the paired non-tumor sample. Statistical data are shown. F. FISH was performed to determine the subcellular distribution of MIR99AHG in A549 cells. ***P < 0.001. [file 12967_2022_3633_MOESM2_ESM.docx]

**Additional figure legends**

**
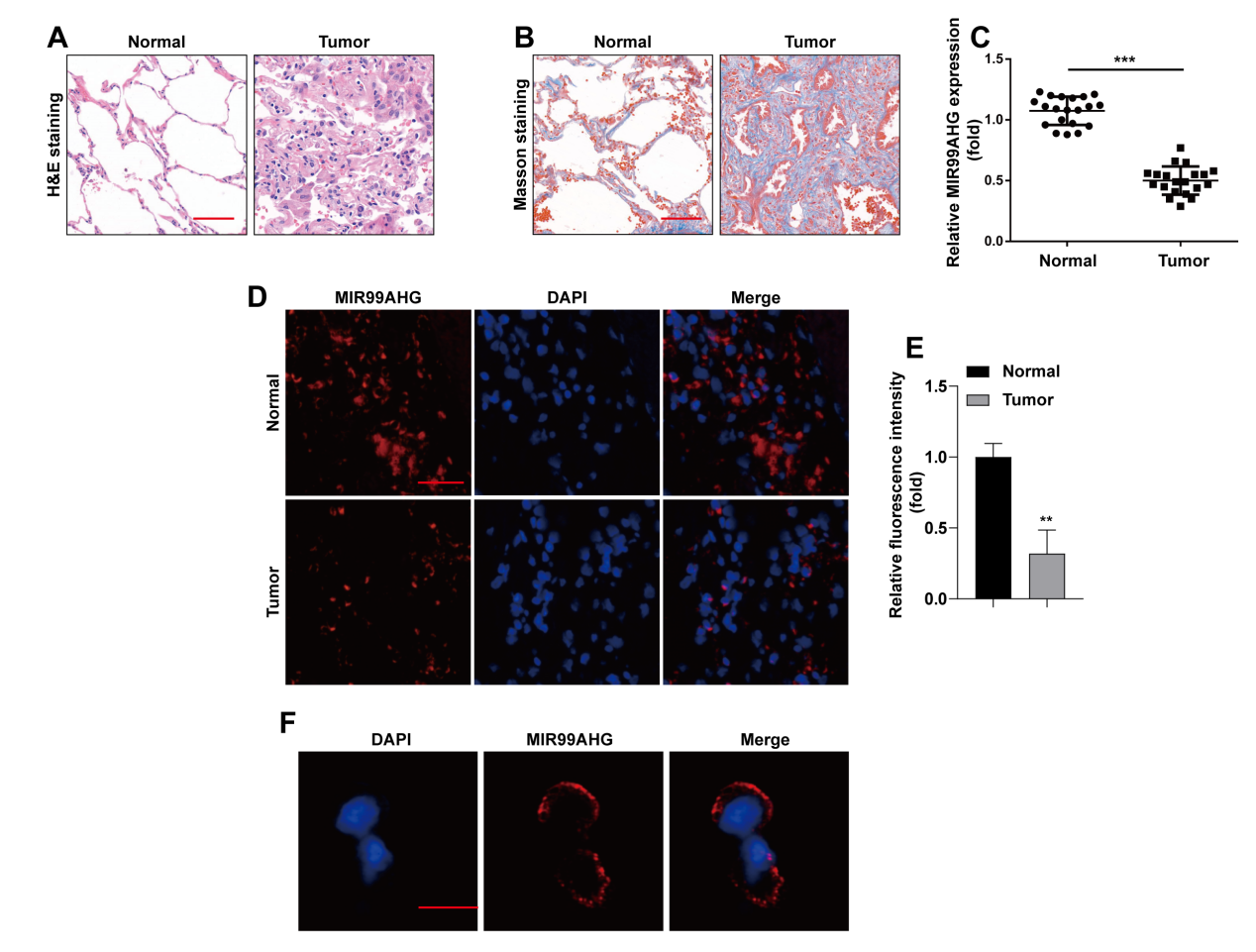
**

**Figure S1. Abnormal expression of MIR99AHG in LUAD tissues. A.** H&E staining of the LUAD tumor tissues and non-tumor tissues. **B**. Masson staining of tissue from LUAD with fibrosis. **C**. The expression levels of MIR99AHG in 20 paired LUAD tissues and non-tumor specimens were determined using RT-qPCR. **D, E.** FISH was used to determine the expression of MIR99AHG in LUAD tissue and the paired non-tumor sample. Statistical data are shown. **F.** FISH was performed to determine the subcellular distribution of MIR99AHG in A549 cells. ****P* < 0.001
